# Cross-Talk between Human Neural Stem/Progenitor Cells and Peripheral Blood Mononuclear Cells in an Allogeneic Co-Culture Model

**DOI:** 10.1371/journal.pone.0117432

**Published:** 2015-02-06

**Authors:** Hongxia Zhang, Bei Shao, Qichuan Zhuge, Peng Wang, Chengcai Zheng, Weilong Huang, Chenqi Yang, Brian Wang, Dong-Ming Su, Kunlin Jin

**Affiliations:** 1 Zhejiang Provincial Key Laboratory of Aging and Neurological Disorder Research, First Affiliated Hospital, Wenzhou Medical University, Wenzhou 35000, China; 2 Department of Pharmacology and Neuroscience, University of North Texas Health Science Center, Fort Worth, TX 76107, United States of America; Georgia Regents University, UNITED STATES

## Abstract

Transplantation of human neural stem/progenitor cells (hNSCs) as a regenerative cell replacement therapy holds great promise. However, the underlying mechanisms remain unclear. We, here, focused on the interaction between hNSCs and allogeneic peripheral blood mononuclear cells (PBMCs) in a co-culture model. We found that hNSCs significantly decrease the CD3^+^ and CD8^+^ T cells, reduce the gamma delta T cells and increase the regulatory T cells, along with reduced pro-inflammatory cytokines and increased anti-inflammatory cytokines after co-culture. We also found that PBMCs, in turn, significantly promote the proliferation and differentiation of hNSCs. Our data suggest that hNSCs cross-talk with immune cells.

## Introduction

Neural stem/progenitor cells (NSCs) derived from human fetal forebrain [1] or embryonic cells [2] have the potential to reconstitute the damaged nervous tissues in some neurological disorders and traumatic nervous system lesion after being transplanted into the brain. The improvement of outcomes and recovery of functional deficits after transplantation of NSCs have been confirmed in numerous animal models for therapeutics of multiple sclerosis, Parkinson’s disease, spinal cord injury, and ischemic stroke [3,4,5,6].

However, one of the major hurdles to cell therapy is how the host’s immune system responds to transplanted cells. More often than not, an immune response is induced thus leading to rejection reactions to allogeneic NSCs [7]. A crucial step therefore, toward efficient clinical application of NSCs transplantation is to reveal the interaction between immune cells and expanded NSCs *in vitro* and *in vivo*. Interestingly, expanded NSCs *in vitro* displayed different levels of the major histocompatibility complex (MHC) class I or class II, most likely owing to different culture conditions and tissue origin [8]. Despite expressing MHC class I and II molecules as well as co-stimulatory molecules, human NSCs were shown not to induce an allogeneic T cell response *in vitro* [8]. Thus, there was still a need to further elucidate the activation and differentiation of human NSCs (hNSCs) in the interaction with peripheral blood mononuclear cells (PBMCs), in which there are many kinds of immune cells. Here, we investigated if NSCs can induce proliferation and differentiation of T lymphocytes *in vitro*.

Conversely, it is also important to know whether T cells, which may potentially affect the function of NSCs in immunomodulation, could in turn influence the maturation, proliferation and differentiation of NSCs. Mezey et al [9] suggested a link between the immune system and survival of NSCs stating that transplanted bone marrow cells could differentiate into mature neurons. The inflammatory response after ischemic stroke is known to induce reparative mechanisms including neurogenesis [10]. Following insult-induced neurogenesis, subsequent events happen including the proliferation of NSCs, migration of neuroblasts, and survival and maturation of neurons *in vivo* [11]. Despite that, the details of this link are still unclear.

In addition to cell replacement therapy, in-depth studies have shown that transplanted NSCs play therapeutic roles through their migration into inflammatory sites and releasing neurotrophic and immunomodulatory factors to interact with immune cells, termed as “chaperone effect” [12,13,14]. Similarly, NSCs exert their beneficial effects not only by cell replacement but also by immunomodulation and trophic support [15]. It was reported that NSCs could inhibit inflammatory responses mediated by interleukin 2 (IL-2) and interleukin 6 (IL-6) [16]. The secretion of transforming growth factor beta (TGF-β) could also down-regulate the proliferative response in mixed lymphocyte reaction (MLR) [17]. However, the current knowledge of underlying mechanisms by which NSCs modulate immune response is still limited.

To identify the cross-talk of hNSCs with immune cells, we examined the fates of NSCs and T cells during co-culture. Our data showed that hNSCs were able to reduce the proportion of CD3^+^ T lymphocytes especially the CD8^+^ and gamma delta T (γδT) lymphocyte subpopulations. We also found that hNSCs could significantly increase the proportion of CD4^+^CD25^+^Foxp3^+^, and alter cytokine profiles involved in immune-modulation and inflammation. In addition, the presence of PBMCs in turn, promoted the proliferation and differentiation of hNSCs.

## Materials and Methods

### Human tissue and cell culture

Brain tissue from human first trimester (6–12 weeks of gestation; n = 12) was obtained following routine abortions by vacuum aspiration as previously described [18]. The complete study was approved by the Human Ethics Committee of the First Affiliated Hospital, Wenzhou Medical University and followed the guidelines of the US Public Health Service, which includes written informed consent from pregnant women. Standard health screens were performed before abortion. The human fetal forebrain tissues were mechanically dissociated under sterilized conditions into small cubes using steriled scissors and repeatedly pipetted using plastic Pasteur pipette in Neurobasal medium (Gibco, USA), then cell suspensions were filtrated through 400 strainer. The filtrated single cells were washed once and immediately seeded in 25 cm^2^ culture flask (1 x10^6^ cells) in NEF medium (consisting of a 1:1 mixture of Dulbecco’s modified Eagle’s medium-F12 (Invitrogen) supplemented with 1% N2 supplement (Invitrogen); 0.5% B27 (Invitrogen); 25 mg/ml insulin (Sigma-Aldrich); 6 mg/ml glucose (Sigma-Aldrich); 5mM HEPES (Invitrogen); 20 ng/ml basic fibroblast growth factor (Sigma-Aldrich) and 20 ng/ml epidermal growth factor (Sigma-Aldrich)) [19]. At the beginning of cells forming aggregates which developed into neurospheres (about 3 days), the cells were collected for use.

### Immunocytochemistry

The neurospheres were confirmed by cell-type-specific markers as described previously [20], and human cell characterization was performed by immunocytochemistry using the following primary antibodies: anti-nestin (1:200; Abcam), anti-sex determining region Y-box 2 (Sox2, 1:50; Cell Signaling Technology), anti-doublecortin (DCX, 1:100; Abcam), anti-Neuron-specific Nuclear Protein (NeuN, 1:100; Millipore), anti-Ki67 (1:400; Cell Signaling Technology), and anti-glial fibrillary acidic protein (GFAP, 1:100; Abcam). Cells were then incubated with the corresponding secondary antibodies for 1 hr at 37°C. Cell nuclei were stained with 4, 6-diamidino-2-phenylindole (DAPI; 5 μg/ml; Sigma).

### Human NSC and immune cell co-cultures

PBMCs were collected from normal adult human peripheral blood (n = 9) with oral consent based on HHS regulations under (45 CFR 46.116(c) or (d)), which was approved by ethics committees of the First Affiliated Hospital. PBMCs isolated by density gradient centrifugation under sterilized conditions. Collected neurospheres were cultured onto PLL-coated cover glasses in 24-well plates at a density of 1.5×10^6^ cells/well in 1 ml of RPMI-1640 complete medium with 5% FBS until cells grew to 60–70% confluence. The ratio of hNSCs:PBMCs in detection of lymphocyte subtype was 1:10, 1:1 and 5:1 respectively and then at a ratio of 5:1 in other co-culture groups. The PBMCs were then loaded for the co-culture with the plated hhNSCs for 2 days. Cultured PBMCs- or hNSPCshNSCs-only were designated as control groups, respectively.

### Flow cytometry analysis

After 2 days co-culture of hNSCs and PBMCs, we aspirated suspensions contained PBMCs using plastic Pasteur pipette and washed cells for use. For cell surface marker staining, the following fluorochrome-conjugated anti-human antibodies were used (all from BD Biosciences): polyethylene (PE) anti-TCRγδ (B1), FITC anti-CD3 (UCHT1), PE-CY5 anti-CD4 (RPA-T4), PE anti-CD8 (RPA-T8). Cells were incubated with antibodies and washed once with PBS before analysis on a BD FACS Calibur flow cytometer. For Treg cell detection, FITC anti-CD4 (RPA-T4), allophycocyanin (APC) anti-CD25 (M-A251) and PE anti-FOXP3 (259D/C7) were used according to manufacturer’s instructions. For cell sorting, stained cells were sorted on a FACS machine (BD Calibur, USA) and the results were analyzed using FlowJo7.6.1 software.

### Proliferation assay

Freshly isolated 10×10^6^ cells/ml of PBMCs were labeled by 5-(and 6)-Carboxyfluorescein diacetate succinimidyl ester (CFSE, Sigma) at a concentration of 10 μM for 15 min at 37°C in the dark. The labeling process was stopped by adding a 5-fold volume of pre-warmed RPMI-1640 media for another 30min, which was followed by a co-culture with hNSCs. After a 48 hr co-culture, the PBMCs were stained with APC anti-CD3 and then analyzed on the FACS Calibur. Similarly, human neurospheres were labeled with CFSE and seeded to the plates for co-culture. After 48 hr, PBMCs were discarded and adherent hNSCs were pipetted for staining with APC anti-CD3.

### Cell counting kit-8 (CCK-8) assay

Viability of hNSCs was assessed with the CCK-8 assay (Dojindo Laboratories, Kumamoto, Japan) in a transwell culture system. Transwell chambers with a 3-μm pore size membrane (Corning Costar) were used to separate the lymphocytes and hNSCs and played a role in the interaction effect of lymphocytes and hNSCs by medium. Briefly, there were 600 μl medium containing suspended PBMCs in the lower chamber and 100μl medium containing hNSCs in the upper chamber. After 2 days of incubation, 600 μl medium in the lower chamber was discarded and 100μl medium in the upper chamber was aspirated gently. Then 300 μl of medium containing 30 μl of CCK-8 (volume ratio 1/10) was poured into the lower chamber which can immersed into the upper chamber, and then the cells were incubated at 37°C for 3 hrs. Cell viability was measured at 450 nm absorbance using a microplate reader (Bio-Tek, USA).

### Apoptosis assay

The percentage of apoptotic T lymphocytes and hNSCs after 2 days of co-culture was evaluated by using Alexa Fluor 488 Annexin V/Dead Cell Apoptosis Kit (Invitrogen). After co-culture, PBMCs and hNSCs were collected respectively, and both were stained with APC anti-CD3, then washed and re-suspended in 1X annexin-binding buffer, 5 μl Annexin V and 1 μl 100 μg/ml Propidium Iodide (PI) working solution for 15 minutes at room temperature. Finally, the cells were analyzed by flow cytometry.

### Western blot analysis

Co-cultured hNSCs were lysed with Protein Extraction Reagent (Biotime) containing Protein Inhibitor Cocktail. Protein concentrations were measured with the BCA kit (Biotime, China). Protein (20 μg) was resolved by sodium dodecyl sufate-polyacrylamide gel electrophoresis (SDS-PAGE) and transferred to a PVDF Membrane. Primary antibodies were anti-nestin (1:100), anti-Sox2 (1:1000), anti-DCX (1:1000), anti-NeuN (1:100), anti-GFAP (1:5000). The secondary antibodies used were horseradish peroxide (HRP)-conjugated goat anti-mouse and goat anti-rabbit IgG (1:10000; Bioworld). Loading control used was monoclonal anti-GAPDH (1:10000; Bioworld) to probe stripped membranes. The signal was developed with chemiluminescence reagents (ECL Advance) and the result was evaluated as a ratio of optical density between the targeted protein band and the GAPDH band.

### Cytometric Bead Array (CBA)

Levels of human Interleukin-2 (hIL-2), hIL-4, hIL-6, hIL-10, human interferon-γ (hIFN-γ), human tumor necrosis factor-α (hTNF-α) and hIL-17 in cell culture supernatant were measured with CBA human Th1/Th2/Th17 Cytokine Kit (BD Biosciences, USA) according to the manufacturer’s instructions. Mixed capture beads were added into each sample, and the Th1/Th2/Th17 PE Detection Reagent was then added into all tests for 3 hrs at room temperature. Similarly, hTGF-β level was measured using the BD CBA Human Soluble Protein Master Buffer Kit (BD Biosciences, USA) according to the manufacturer’s instructions. Beads were analyzed on the FACS Calibur.

### Statistical analysis

Data are expressed as the mean ± standard error of the mean (SEM). Statistical comparisons were performed by two-way analysis of variance (ANOVA) with repeated measures, followed by *post hoc* multiple comparison tests (Fisher PLSD or Student’s paired *t* test with the Bonferroni correction). A *p*-value of less than 0.05 was considered to be statistically significant. At least three NSC-PBMC culture pairs were evaluated for each group, and the experiments were repeated at least 3 times for each experiment.

## Results

### Derivation and characterization of hNSCs

To confirm successful isolation of hNSCs, the morphology of neurospheres and surface markers of hNSCs were determined. We found that aggregates of dividing cells derived from the human fetal forebrain could form neurospheres (about 3 days) and displayed small bright spheres (S1A Fig.), which share some similar features with hNSCs. In addition, immunostaining showed that these cells were NSC protein markers (DCX, nestin and Sox2), and proliferative marker (Ki67) (S1B and S1C Fig.). Only a small portion of these cells expressed mature neuron cell marker NeuN (<10%) and astrocytes marker GFAP (<5%). Only a few of apoptotic cells in hNSCs were observed when the cells were cultured alone.

### Effect of hNSCs on lymphocytes

**Fig 1:**
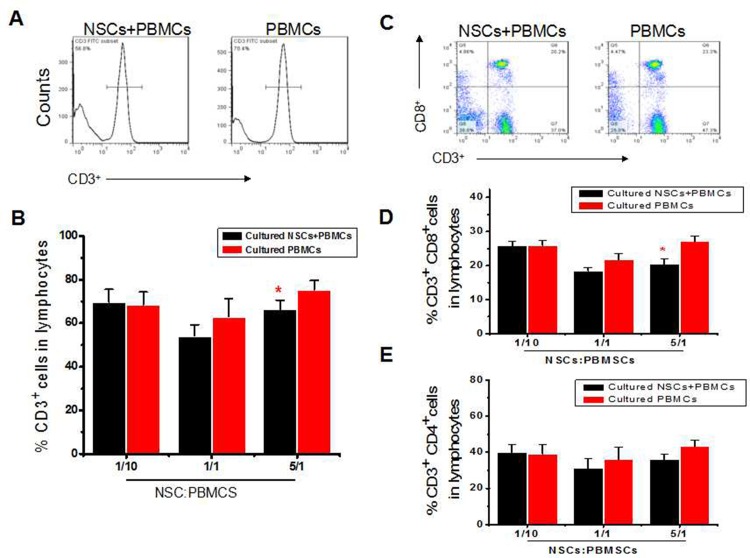
Effects of hNSCs on proportions of T cell proportion (CD3^+^), CD8^+^ T cell proportion (CD3^+^CD8^+^) and CD4^+^ T cell proportion (CD3^+^CD4^+^). (**a**). Representative histogram of T cell proportion (CD3^+^). (**b**). Statistical analysis of T cell proportion. (**c**). Representative dot plots of CD8^+^ T cell proportion (CD3^+^CD8^+^). (**d**). Statistical analysis of CD8^+^ T cell proportion. (**e**). Statistical analysis of CD4^+^ T cell proportion. *, *P* < 0.05. The experiments were repeated at least 3 times for each experiment.

**Fig 2:**
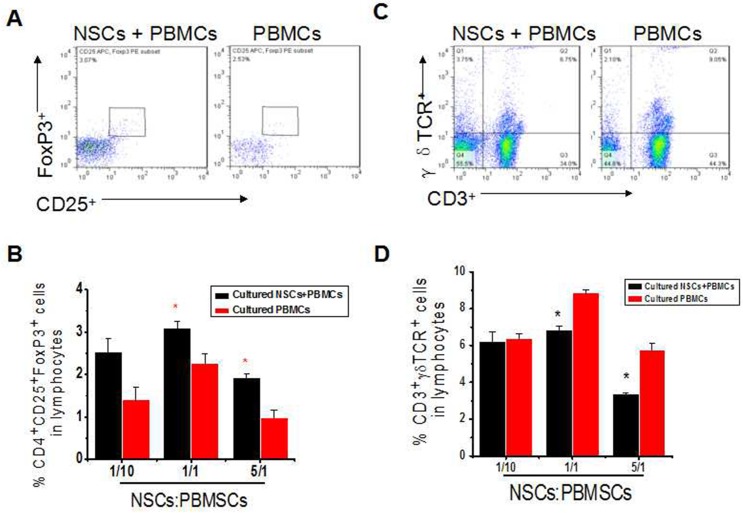
Effects of hNSCs on proportions of γδT cells and Tregs. (**a**). Representative dot plots of Treg proportion (CD4^+^CD25^+^Foxp3^+^). (**b**). Statistical analysis of Treg proportion. (**c**). Representative dot plots of γδT cell proportion (CD3^+^γδTCR^+^). (**d**). Statistical analysis of γδT cell proportion. *, P < 0.05. The experiments were repeated at least 3 times for each experiment.

Evidence indicates that hNSC transplantation can improve the outcomes after focal cerebral ischemia in rodents [21]. Therefore, we investigated whether the hNSCs were able to suppress a response in allogeneic T lymphocytes *in vitro*. After co-culture of hNSCs with PBMCs at a ratio of 1:10, 1:1 and 5:1 for 48 hrs, we found that hNSCs significantly decreased the CD3^+^ cells (Fig. 1A and 1B), and CD8^+^ T cells in particular (Fig. 1C and 1D), but CD4^+^ T cells were less affected (Fig. 1E) at the 5:1 ratio, compared to PBMCs cultured alone. Notably, no significant difference was observed when hNSCs were co-cultured with PBMCs at a ratio of 1:10 and 1:1. This was consistent with the results of previous findings obtained by Julia Knight [22]. In addition, γδT cells were found to be decreased profoundly after co-culture with hNSCs compared to control group (Fig. 2C and 2D).

To further study if other immune cell subsets were influenced by hNSCs, hNSCs and PBMCs were co-cultured at a ratio of 1:10, 1:1 and 5:1 for 48 hr. We found that hNSCs robustly promoted an increase in the proportion of Tregs, when hNSCs were co-cultured with PBMCs at a ratio of 5:1 and 1:1, but not 1:10, compared to PBMCs-only group (Fig. 2A, 2B).

**Fig 3:**
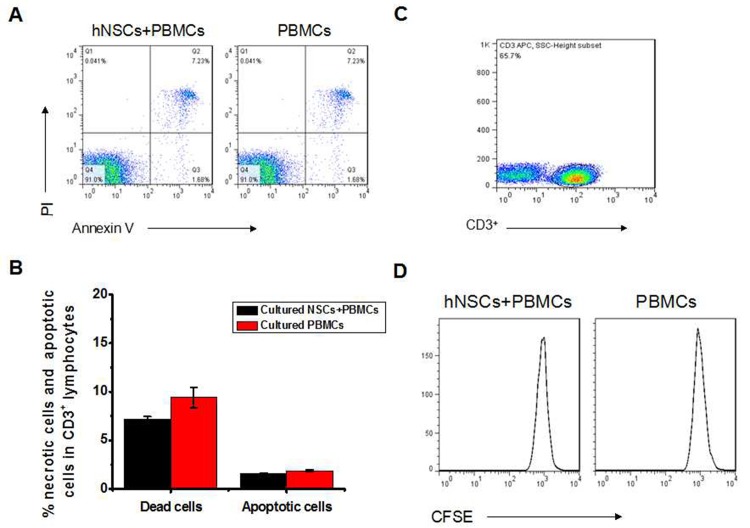
Effects of hNSCs on apoptosis and proliferation of T cells. (**a**). Representative dot plots of apoptotic T cells (Annexin V and PI) after co-culture with hNSCs (1:5). (**b**). Statistical analysis of necrotic and apoptotic T cells. (**c**). Representative dot plots of T cell proportion (CD3+). (**d**). Representative histogram of proliferation of T cell proportion (CFSE). The experiments were repeated at least 3 times for each experiment.

To determine whether apoptosis or cell death occurred, PBMCs were stained with Annexin/V and PI after co-culture at a ratio of 5:1 for 48 hrs. We found no significant differences in apoptotic and dead cells between co-culture and control groups (Fig. 3A, 3B).

To determine whether hNSCs induced or elicited a proliferative response in allogeneic T cells *in vitro*, we analyzed NSC-induced expansion with CFSE-labelled PBMCs after hNSCs were co-cultured with PBMCs at a ratio of 5:1 for 48 hrs. We found that neither proliferative nor suppressive responses in PBMCs occurred after co-culture with NSCs, compared to PBMCs-only group (Fig. 3C, 3D).

### Cytokines in the cell supernatant

**Fig 4:**
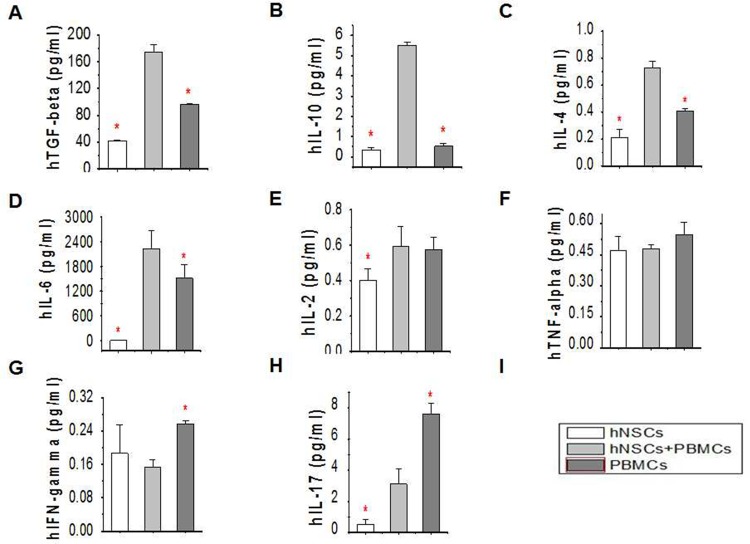
Effects of NSCs on cytokine profiles. Human NSCs were co-cultured with human PBMCs for 2 days. TGF-β (A), IL-10 (B), IL-4 (C), IL-6 (D), IL-2 (E), TNF-α (F), INF-γ (G) and IL-17 (H) levels in the supernatant were determined. *, P < 0.05, compared with co-culture group. The experiments were repeated at least 3 times for each experiment.

Next, we asked whether hNSCs affected the levels of pro- and anti-inflammatory cytokines. hNSCs were co-cultured with PBMCs at a ratio of 5:1 for 48 hrs, and then the levels of hIL-2, hIL-4, hIL-6, hIL-10, hIFN-γ, hTNF-α and hIL-17 in the supernatants were measured using the CBA human Th1/Th2/Th17 Cytokine Kit. We found that anti-inflammatory cytokines, TGF-β, IL-10 and IL-4, were apparently increased and pro-inflammatory cytokines, IFN-γ and IL-17, were significantly decreased, compared to cultured PBMCs- or hNSCs-only groups (Fig. 4A-C). TNF-α and IL-2 were not affected (Fig. 4E-H). Interestingly, the IL-6 secretion was higher in co-culture supernatant than control groups.

### Effect of lymphocytes on hNSCs

**Fig 5:**
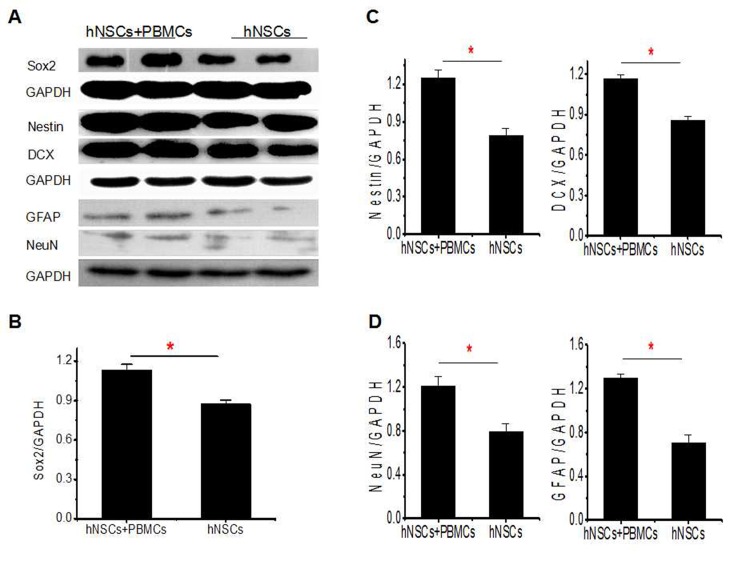
Effects of PBMSCs on differentiation of hNSCs. (**a**). Representative bands of Sox2 and GAPDH. (**b**). Statistical analysis of Sox2 (Sox2/GAPDH). (**c**). Representative bands of Nestin, DCX and GAPDH. (**d**). Statistical analysis of Nestin (Nestin/GAPDH) and DCX (DCX/GAPDH). (**e**). Representative bands of GFAP, NeuN and GAPDH. (**f**). Statistical analysis of GFAP (GFAP/GAPDH) and NeuN (NeuN /GAPDH).*, P < 0.05. The experiments were repeated at least 3 times for each experiment.

Finally, we asked if the fate of the hNSCs could be influenced by PBMCs. To identify the differentiation of NSCs in the presence of PBMCs (5:1), Western blot analysis was performed to analyze the expression levels of nestin, Sox2, DCX, NeuN and GFAP. As shown in Fig. 5A-D, the expression levels of Sox2, nestin, DCX, NeuN and GFAP were upregulated in the co-culture group, compared to control groups.

**Fig 6:**
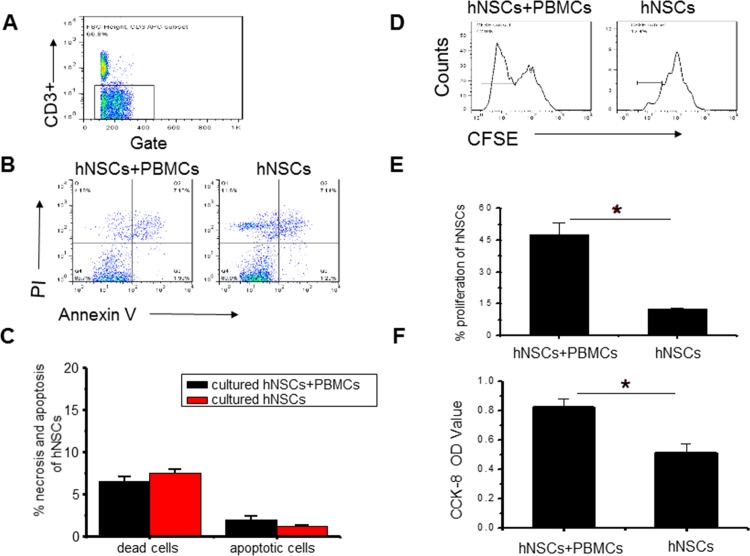
Effects of PBMCs (5:1) on apoptosis and proliferation of NSCs. (**a**). Representative dot plots of T cell proportion (CD3+). (**b**). Representative dot plots of apoptotic NSCs (Annexin V and PI). (**c**). Statistical analysis of necrotic and apoptotic cells. (**d**). Representative histogram of proliferation of NSCs (CFSE). (**e**). Statistical analysis of proliferation of NSCs (CFSE). (f). Statistical analysis of proliferation of NSCs (CCK-8). *, P < 0.05. The experiments were repeated at least 3 times for each experiment.

To assess the apoptosis or death of hNSCs, hNSCs with Annexin/V and PI were stained after co-culture at a ratio of 5:1 for 48 hrs. We found no significant differences between the two groups (Fig. 6A-C). Next, we determined whether the proliferation of hNSCs was influenced by PBMCs. PBMCs were co-cultured with CFSE-labelled hNSCs in 24-well plate for 48 hrs and the presence of PBMCs apparently stimulated the proliferation of hNSCs (Fig. 6D-E). Consistent with the CFSE result, the evaluation of CCK-8 in a transwell culture system also showed there was a notable proliferation of hNSCs when hNSCs were co-cultured with PBMCs, compared to control group (Fig. 6F).

## Discussion

In this study, we found evidence of the direct interaction between hNSCs and peripheral T lymphocytes. Firstly, hNSCs profoundly affected total CD3^+^ T cells, in particular, subpopulations of CD8^+^ T cells, γδT cells, and Tregs, along with decreased pro-inflammatory cytokines and increased anti-inflammatory cytokines, implying that hNSCs were able to modulate inflammatory responses induced by T cells *in vitro*. Secondly, the PBMCs in turn affected the proliferation and differentiation of hNSCs. Our data suggest that the immunomodulatory capacity of hNSCs is likely derived from their ability to selectively delete deleterious and support protective actions of inflammatory cells and cytokines.

Our data was consistent with previous findings by Julia Knight [22], which suggested that neural stem/progenitor cells can specifically reduce pro-inflammatory T subtypes in a contact-dependent manner and illuminated the immunomodulatory capacity of NSCs. In addition, we found that at both 5:1 and 1:1 ratio, γδT cells were profoundly decreased after co-culture with hNSCs compared to control group (Fig. 2C and 2D). Several studies confirm that CD3^+^ T are involved in infiltration of inflammation [23,24] and γδT cells, which are a major source of IL-17 production *in vitro* [25], induces neuronal damage in the brain [26]. No doubt, decrease of total CD3^+^ and γδT cells reduces neuronal damage.

T cells, particularly CD8^+^ and γδT cells, are known to be critical T cell subsets associated with detrimental effects, while CD4^+^ Th2 and CD4^+^ Treg cells are associated with protective effects in the surrounding neural parenchyma after cerebral ischemia or degenerative diseases upon activation [27]. Some reports proposed that the expression of MHC class molecules in NSCs can be strongly up-regulated by pro-inflammatory cytokines *in vivo* [28], whose immunogenicity can induce proliferative responses in T lymphocytes. Despite that, hNSCs share similar immunosuppressive characteristics with BMSCs and are known to have strong immune-suppressive properties on all cells of the immune system [29]. Of note, we found that hNSCs did not induce the apoptosis but rather delete the proportion of total CD3^+^ T cells (mostly CD8^+^ subset) in a dose-dependent manner, showing immune inhibitory effect by hNSCs, consistent with results defined by Li [30] and Liu [31]. Although the percentage of CD4^+^ T cells significantly increased in the cell–cell contact system in the presence of hNSCs for 3 days[31], increased CD4^+^ T cells were likely Treg cells. This difference may be due to the different co-culture duration or other factors such as pre-incubation with IFN-γ and different immunogenicity of hNSCs with regards to its derivation of tissue and state of differentiation [32].

Furthermore, Tregs appear to be good candidates for cellular therapy, as they can prevent development of autoimmune diseases, tumor immunity, graft rejection, and graft-vs-host disease [33]. Emerging evidence suggests that Tregs are involved in immunomodulation following mesenchymal stem cell (MSC) infusion [34]. It has been proved that Tregs can prevent secondary injury expansion and the depletion of Tregs increased delayed brain damage and deteriorated functional repair [35]. As for γδT cells, expanded γδT cells have been previously reported to exert a strong cytotoxic activity to plasma cell and melanoma cell lines [36]. Based on the therapy of cell transplantation, IL-17-inducing γδT cells have been implicated in the pathogenesis of inflammatory lesions, in which Th1 cytokines and cytotoxic factors are thought to play a critical role [37]. Our data now show that hNSCs can induce an increase of Tregs and a dramatic reduction of γδT cells. These findings suggest that hNSCs can modulate T cell subpopulations as shown in our previous published *in vivo* data[24].

Finally, our results show that hNSCs had no influence over the apoptosis of T cells when co-cultured, which is consistent with Einstein’s work, showing that hNSCs do not directly induce T cell apoptosis, but rather inhibit T cell activation in a non-specific manner [38]. Similarly, we found that PBMCs did not exert any influence over the apoptosis of hNSCs in the in a 2 day co-culture. Various groups have reported that PBMCs have an effect on the apoptosis of hNSCs and attributed it to the ratio of hNSCs vs. PBMCs, the differences of medium, manner, and co-culture durations. Further investigation into this phenomenon should be performed.

hNSCs of fetal origin or derived from human ESC have the potential to differentiate into mature neurons after transplantation into the CNS[32]. Others have confirmed that endogenous neurogenesis in the hippocampus is suppressed by lipopolysaccharide-induced inflammation [39,40], which means that the suppression of immune response can promote neuronal differentiation [41]. Our data show that PBMCs affects hNSC proliferation and differentiation after their co-culture, where levels of hNSC special surface markers were increased, and so did the number of mature neurons and astrocytes. This phenomenon may be related to the suppression of immune response and the release of cytokines. Among those cytokines, IL-6 appears to play an important role in the fate of hNSCs [39]. It was reported [42] that the proliferation of microglials promoted by hNSCs was involved in the increasing secretion of IL-6, which can, in turn, influence microglials in phenotype. Ideguchi [41] and Gomi [43] found that the IL-6 decreased the neuron:astrocyte ratio, and showed that the neutralizing antibody against IL-6 was able to abolish this effect. These all suggest that the proliferation of microglial cells is related to the increased levels of IL-6[41]. Our results show increased IL-6 during co-culture of hNSCs with PBMCs and increased expression of GFAP, which is the surface marker of astrocytes, implying that there is a positive relationship between the increased level of IL-6 and the proliferation of astrocytes. TNF-α and IFN-γ have been shown to have dual effects on neurogenesis and neuronal differentiation [44,45], and our data showing the decrease of IFN-γ in co-cultures may also explain the differentiation of hNSCs. The fact that hNPCs can migrate into the damaged CNS [46,47] together with their chronic presence within the brain tissue may enhance neuroprotective effects and make cell transplantation therapies possible.

Our data suggested that T cells affect proliferation of NSCs. However, the underlying mechanisms remain unclear. Some studies indicate that hNSCs suppress the activation of human T cells through cell to cell physical contact [14,22]. Other studies indicated that hNSCs exert their modulatory effects by secreting an array of immunomodulatory cytokines and trophic support. The release of IL-6 can suppress neuronal differentiation and promote glial cell fate[43] and upregulate other primary pro-inflammatory cytokines (for example, TNF-α, IL-2, IFN-γ) thus rendering transplanted hNSCs being unable to fully differentiate[48], while TGF-β and IL-10 secreted by hNSCs are considered to be neuroprotective [49]. Whatever the underlying mechanism, it is now confirmed that hNSCs not only form neural cells for replacement, but also produce immunomodulatory and trophic molecules capable of promoting tissue repair [12]. Our *in vitro* data show that anti-inflammatory cytokines TGF-β, IL-10, and IL-4 were apparently increased and pro-inflammatory cytokines IFN-γ and IL-17 were reduced in the co-cultures. In addition, the levels of IL-6 were higher than control groups, which might be related to the differentiation of hNSCs. Inflammatory responses that take place following transplantation of stem cells after ischemia mostly involved immune cells like microglial cells and astrocytes [50]. These microglial cells are activated to secrete immune mediators, among which both astrocytes and microglial cells can secrete IL-6, IL-1β, TNF-α, and other chemotactic factors [50]. Although it was mostly reported that the level of IL-6 was decreased after cell transplantation or in co-cultures involving stem cells and T cells, there was a recent study that showed the increase of IL-6 release in co-cultures or microglial mono-cultures than hNSC mono-cultures [42]. This increased IL-6 release could be partially explained by the proliferation of astrocytes in co-cultures. Moreover, other factors that we did not investigate may also play vital roles in the process of immunomodulation. The production and release of brain-derived neurotrophic factor (BDNF) by NSCs could also contribute to increased neuronal viability and improved neuro-functional deficits [51].

## Conclusions

Taken together, our results demonstrated that hNSCs exert the character of suppression on inflammation by ridding detrimental and supporting beneficial inflammatory cells and mediators. Moreover, the suppression of inflammatory reactivity could promote neurogenesis and astrocytogenesis, and this result could be linked with the findings reported by Makoto [41]. Our previous *in vivo* study showed that transplantation of hNSCs improved outcomes and promoted recovery of neuronal function from ischemia [21]. Although hNSCs suppress inflammation and enhance neuronal viability to benefit function of injured CNS both *in vitro* and *in vivo*, the underlying mechanisms of hNSC immunomodulation thus need further studying.

## Supporting Information

S1 Fig Cultured neural cells show neural stem/progenitor characteristics. **(a)**. Neurospheres (about 3 days) derived from fetal forebrain displayed small bright spheres. **(b)**. Expression of neural stem/progenitor, but not mature neural protein markers in culture cells. **(c)**. SOX2-postive cells expressed nestin *in vitro*.
(TIF)
