# Supplementary figures and images for: Cross-Talk between Human Neural Stem/Progenitor Cells and Peripheral Blood Mononuclear Cells in an Allogeneic Co-Culture Model
Source: PLoS One. 2015 Feb 6;10(2):e0117432. doi: 10.1371/journal.pone.0117432 (PMC4319716; doi:10.1371/journal.pone.0117432)

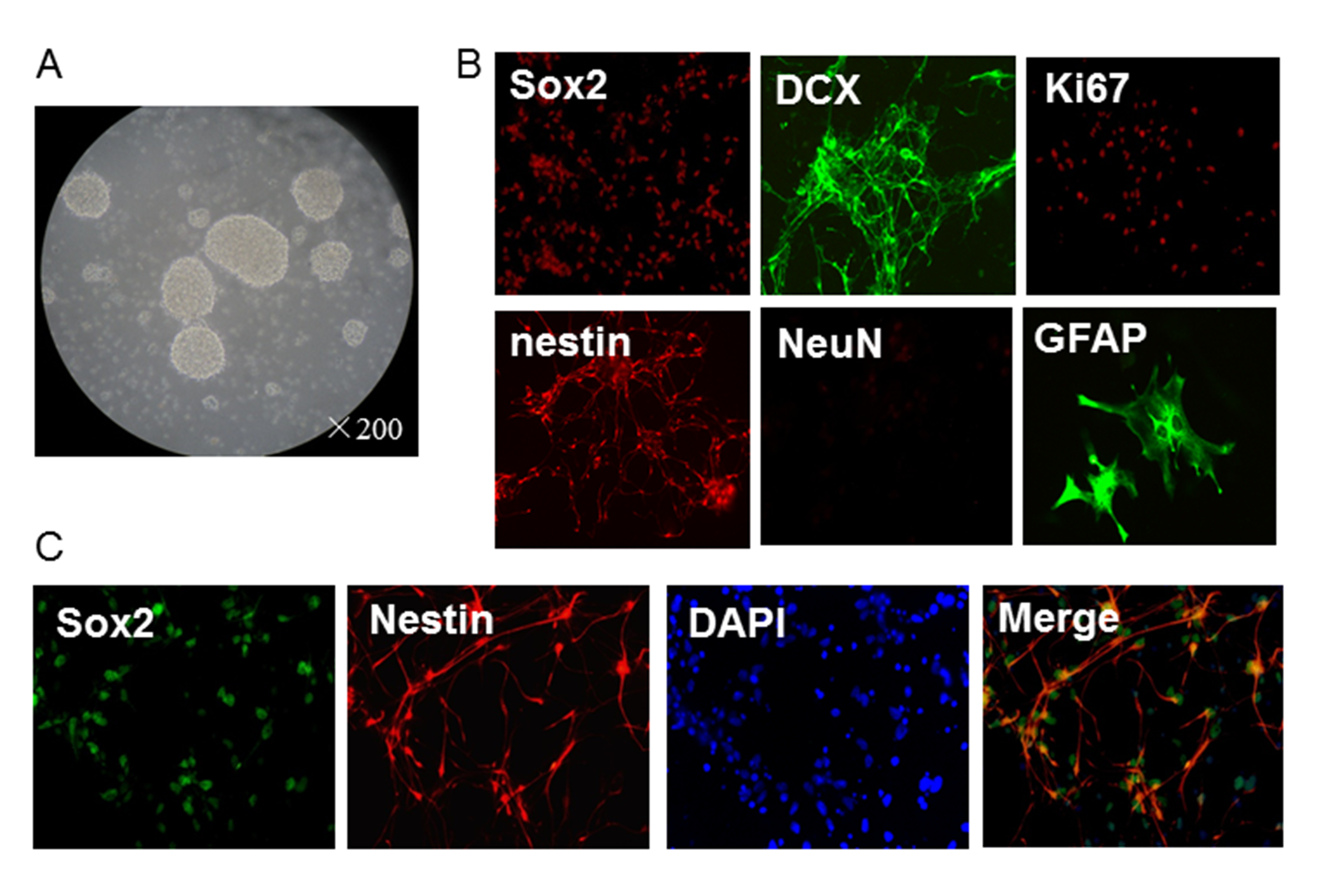

Supplement: S1 Fig — (a). Neurospheres (about 3 days) derived from fetal forebrain displayed small bright spheres. (b). Expression of neural stem/progenitor, but not mature neural protein markers in culture cells. (c). SOX2-postive cells expressed nestin in vitro. (TIF) [file pone.0117432.s001.tif]
